# Organon Society of Boston

**Published:** 1889-05

**Authors:** S. A. Kimball

**Affiliations:** Secretary


### ORGANON SOCIETY OF BOSTON, MASS.

### Meeting Febuary 7th, 1889.

On account of the absence of Dr. Wesselhœft, Dr. Kennedy read, beginning at Section 100.

Dr. Kennedy—In regard to Sections 101 and 102, they seem to me to state a very important feature of Homœopathy. How many physicians realize the value of looking carefully at the first two or three cases of an epidemic; if they did it might save them much trouble later.

Dr. Lincoln—Did Hahnemann express himself as he really meant? He speaks of specifics for epidemics. When speaking of the cholera, he said that two or three remedies would probably cover the majority of the cases.

Dr. Kennedy—I do not think he speaks of a specific remedy, but of the appropriate homœopathic remedy.

Dr. Bell—We must first ascertain what Hahnemann means. I think he refers to such epidemics as do not always retain characteristics, and would therefore exclude yellow fever, smallpox, etc. How much then will it exclude and include? I have always thought it applied to epidemics of influenza or winter diarrhœa, and these may differ in January, February, or March. I have tried to apply Hahnemann's teachings, and have seen evidences of the truth. Through last December we had coughs that called for Arnica, causing pain in the sides with desire to hold on. It was quite a severe cough, and, while Arnica did not help every case, it was the remedy for the majority of them. The winter diarrhœas have been characterized by vomiting with stool. Several cases called for Dulcamara. In one case Dulc. did not seem indicated as well as some other remedy, but that producing no result, it was much helped by Dulc.

Dr. Wheeler—I have had one case of diarrhœa this winter which was cured by Dulc.

Dr. Bell—We rarely use Dulc. in winter, but this winter has been rather warm and damp. Section 103 indicates the way Hahnemann got the picture of psora and discovered the antipsorics.

Dr. Lincoln—Can you find a case that represents psora alone or syphilis or sycosis alone? There always seems to be a combination of two or more.

Dr. Bell—"Material," in Section 105, should be "instruments," as in the original.

Dr. Hastings—This should be understood thoroughly, as most of the so-called homœopathic physicians claim that the law is not always sufficient.

Dr. Bell—We should not search for some occult meaning in Hahnemann's language. It seems to me that he means we will find remedies for almost all our cases. We may fail to find the remedy for some case, for the remedy may not be known. He is talking now of the instruments, not of the law governing their use.

Dr. Davis—Does Section 108 exclude symptoms observed on the sick?

Dr. Bell—No, but that is left for masters in the art of observing. See Section 142.

Adjourned to February 28th.

---

### Meeting February 28th.

Dr. Wesselhœft still being absent, Dr. Bell read, beginning at Section 110.

Dr. Bell—Hahnemann is bringing out the theme expressed in Section 110, everywhere in the *Organon*. He has but two or three themes in the *Organon*, and is constantly referring to them. People often ask if "that little sugar" can do them any good. I usually tell them that a powder of Arsenic or Tartar Emetic would look exactly the same as the sugar powder, and that they cannot tell by the looks or the taste of drugs, but by the effect of the remedy after taking it.

Dr. Cobb—I usually ask them if they know what gave them typhoid fever.

Dr. Dutton—Do you use pellets moistened with alcohol for placebos?

Dr. Bell—I generally use dry pellets or powders with pellets in them. Sometimes I use tablets. In regard to Section 111, it is a very common impression that you can make the remedy suit by changing the potency, whereas no potency of a non-indicated drug is of any value.

Dr. Cobb—A friend who is a physician "out West" told me of a man who came to her one afternoon with a raging toothache. She gave him a remedy, and it helped him so much that the next morning he thought he would take home with him what was left of the solution—he lived some distance away, and happened to be in the town for the night. So he put the solution in an essence of peppermint bottle, and successfully relieved himself of other attacks of toothache, and some of his friends also. The doctor knew of these facts by his bringing back the bottle to be filled again, and it was very strong of the peppermint then. The remedy was Merc. sol.$^{200}$.

Dr. Bell—I have known a remedy to act perfectly when given to a man with his mouth full of chewing tobacco. What I gather from Section 117 is that, although few symptoms may be produced in some powders, the drug is just as valuable.

Dr. Eaton—Note 92; will only one drug be indicated in a given case?

Dr. Bell—One will certainly be more appropriate than the others.

Section 121—Dr. Bell—I do not think this was Hahnemann's latest thought. Lyc., Calc., Sil., Nat. mur. are certainly better proved in the potencies.

Section 129—Dr. Bell—Hahnemann says additional pellets may be taken. Have any of you seen any difference in the number of pellets given? I have not had much experience in proving, but in prescribing I never noticed that the number of pellets given made any difference.

Dr. Dutton—Are people ever injured by proving?

Dr. Bell—I do not think there is any permanent injury. Provers distinguish the symptoms of the drug from those of ordinary health.

When I was proving Calcarea fluorica, I had a backache from riding that I had never experienced before. I had ridden a great deal, but never had such a backache. Then another feeling that the remedy brought out was the fear of want; the feeling that I should come to want. I knew these symptoms as being entirely different from my feelings in ordinary health, as they were not natural to me.

Adjourned to March 14th. S. A. Kimball, *Secretary.*

